# Impact of antifungal treatment in ICU patients with Candida colonization: analysis of the EPIC II study population

**DOI:** 10.1186/cc10666

**Published:** 2012-03-20

**Authors:** D Kett, G Dimopoulus, E Azoulay, P Echeverria, C De La Cuesta, JL Vincent

**Affiliations:** 1 University of Miami Miller School of Medicine, Miami, FL, USA; 2 University Hospital ATTIKO Medical School, University of Athens, Greece; 3 St-Louis Hospital and Paris VII University, Paris, France; 4 Erasme University Hospital, Université Libre de Bruxelles, Belgium

## Introduction

We wished to evaluate the impact of receiving antifungal therapy in ICU patients with Candida colonization.

## Methods

EPIC II recruited 1,265 ICUs in 76 countries. Patient characteristics were collected on the study day. Outcome data were assessed at ICU and hospital discharge. Patients colonized with Candida spp. were classified as having received antifungal treatment or not (*P < 0.05 compared between groups). Numerical values are reported as mean ± SD and length of stay (LOS) data as median (IQR).

## Results

A total of 13,796 adult patients were in participating ICUs on the study day. Of these, 371 were classified as colonized. Differences in patient characteristics and outcomes are reported (Table 1). Baseline characteristics were similar in colonized patients treated with antifungal therapy compared to those that were untreated. Only a modest difference in the length of stay in the ICU prior to study day (25 (14, 40) vs. 21 (8, 43)) and utilization of mechanical ventilatory support (76% vs. 63%) was noted in the treated compared to the untreated patients with Candida colonization (*P *< 0.05). Despite the relatively similar baseline characteristics and equivalent severity of illness scores, treated patients had an increased ICU (35.3 vs. 22.3%) and hospital (41.0 vs. 27.7%) mortality (*P *< 0.05).

**Table 1:** Patients with Candida colonization: characteristics and outcomes

	Therapy (*n *= 184)	No therapy (*n = *175)
SAPS II	39 ± 15	41 ± 18
SOFA	7.6 ± 4.1	7.4 ± 4.4
MV	76%	63%
Pressor	36%	32%
ICU mortality	35%	22%
Hospital mortality	41%	28%

## Conclusion

As colonized patients receiving antifungal treatment had significantly higher mortality, our data do not support the routine use of antifungal therapy in ICU patients based solely on colonization.
